# AMP-Activated Protein Kinase Regulates Oxidative Metabolism in *Caenorhabditis elegans* through the NHR-49 and MDT-15 Transcriptional Regulators

**DOI:** 10.1371/journal.pone.0148089

**Published:** 2016-01-29

**Authors:** Elizabeth Moreno-Arriola, Mohammed EL Hafidi, Daniel Ortega-Cuéllar, Karla Carvajal

**Affiliations:** 1 Laboratorio de Nutrición Experimental, Instituto Nacional de Pediatría, Mexico City, Mexico; 2 Departamento de Biomedicina Cardiovascular, Instituto Nacional de Cardiología, Mexico City, Mexico; Université catholique de Louvain, BELGIUM

## Abstract

Cellular energy regulation relies on complex signaling pathways that respond to fuel availability and metabolic demands. Dysregulation of these networks is implicated in the development of human metabolic diseases such as obesity and metabolic syndrome. In *Caenorhabditis elegans* the AMP-activated protein kinase, AAK, has been associated with longevity and stress resistance; nevertheless its precise role in energy metabolism remains elusive. In the present study, we find an evolutionary conserved role of AAK in oxidative metabolism. Similar to mammals, AAK is activated by AICAR and metformin and leads to increased glycolytic and oxidative metabolic fluxes evidenced by an increase in lactate levels and mitochondrial oxygen consumption and a decrease in total fatty acids and lipid storage, whereas augmented glucose availability has the opposite effects. We found that these changes were largely dependent on the catalytic subunit AAK-2, since the *aak-2* null strain lost the observed metabolic actions. Further results demonstrate that the effects due to AAK activation are associated to SBP-1 and NHR-49 transcriptional factors and MDT-15 transcriptional co-activator, suggesting a regulatory pathway that controls oxidative metabolism. Our findings establish *C*. *elegans* as a tractable model system to dissect the relationship between distinct molecules that play a critical role in the regulation of energy metabolism in human metabolic diseases.

## Introduction

Cellular energy balance is maintained by several cell signaling networks that regulate the uptake, storage and utilization of nutrients. Defects in maintaining energy homeostasis may lead to metabolic diseases, such as obesity, metabolic syndrome and type II diabetes [1]. Since many key signaling mechanisms in mammals appear to be conserved in the nematode *Caenorhabditis elegans*, this free-living worm has been used as an experimental model to explore the genetic basis of energy metabolism [2–5]. The AMP-activated protein kinase (AMPK), Sterol Regulatory Element-binding Protein (SREBP), Peroxisome Proliferator-Activated Receptor alpha (PPARα) and Peroxisome Proliferator-Activated Receptor-gamma Coactivator 1 alpha (PGC-1α) are some of the signaling molecules that have orthologs in *C*. *elegans* (AAK, SBP-1, NHR-49 and MDT-15, respectively) [6–9].

AMPK is a heterotrimeric protein complex (composed of the catalytic α subunit and the regulatory β and γ subunits [10]) and a conserved fuel-sensing enzyme. Its activation under stress conditions (AMP/ATP ratio increased) essentially drives catabolic processes that yield ATP and restore the cellular energy state [11]. The *C*. *elegans* genome encodes the *aak-1* and *aak-2* genes, which are homologs of the α-catalytic subunits of mammalian AMPK [12]. The AAK-2 subunit becomes phosphorylated at threonine 243 (Thr243), equivalent to Thr172 in the mammalian ortholog [13], and primarily takes part in its activity responding to oxidative stress and controlling survival [12,14]. Physiological (dietary restriction) or pharmacological (metformin or resveratrol) AAK-2 activation increases the longevity and lifespan of *C*. *elegans* and it has been suggested that AAK-2 can additionally [12,15–20] regulate lipid mobilization through the adipose triglyceride lipase enzyme (ATGL-1) to maintain dauer larvae survival [21]. SBP-1 promotes fatty-acid homeostasis by regulating the expression of lipogenic enzymes [6], whereas NHR-49 and MDT-15 activate the expression of genes involved in fatty-acid β-oxidation, transport, elongation and desaturation [8,22,23].

Given the essential role of AAK and SBP-1 and NHR-49 transcriptional factors and MDT-15 transcriptional co-activator in oxidative metabolism and lifespan of *C*. *elegans*, it is plausible to suggest that these regulatory elements form a common axis responding to energy requirements acting to restore energy supply in the whole organism, as has been proposed for mammals [10,24]. To gain further insights on this idea, we assessed the significance of AAK activation in the regulation of lipid and carbohydrate metabolism in *C*. *elegans*. We report that AAK activation modified lipid and carbohydrate oxidative fluxes and that these changes were associated with the transcriptional regulators SBP-1, NHR-49 and MDT-15. Our data suggest that these regulatory mechanisms are conserved between *C*. *elegans* and mammals. Therefore, we propose the use of *C*. *elegans* as a model for the study of human diseases associated with disorders of energy metabolism, such as obesity and type II diabetes.

## Results

### Time-dependent phosphorylation of AAK by AICAR and metformin exposure in *C*. *elegans*

**Fig 1:**
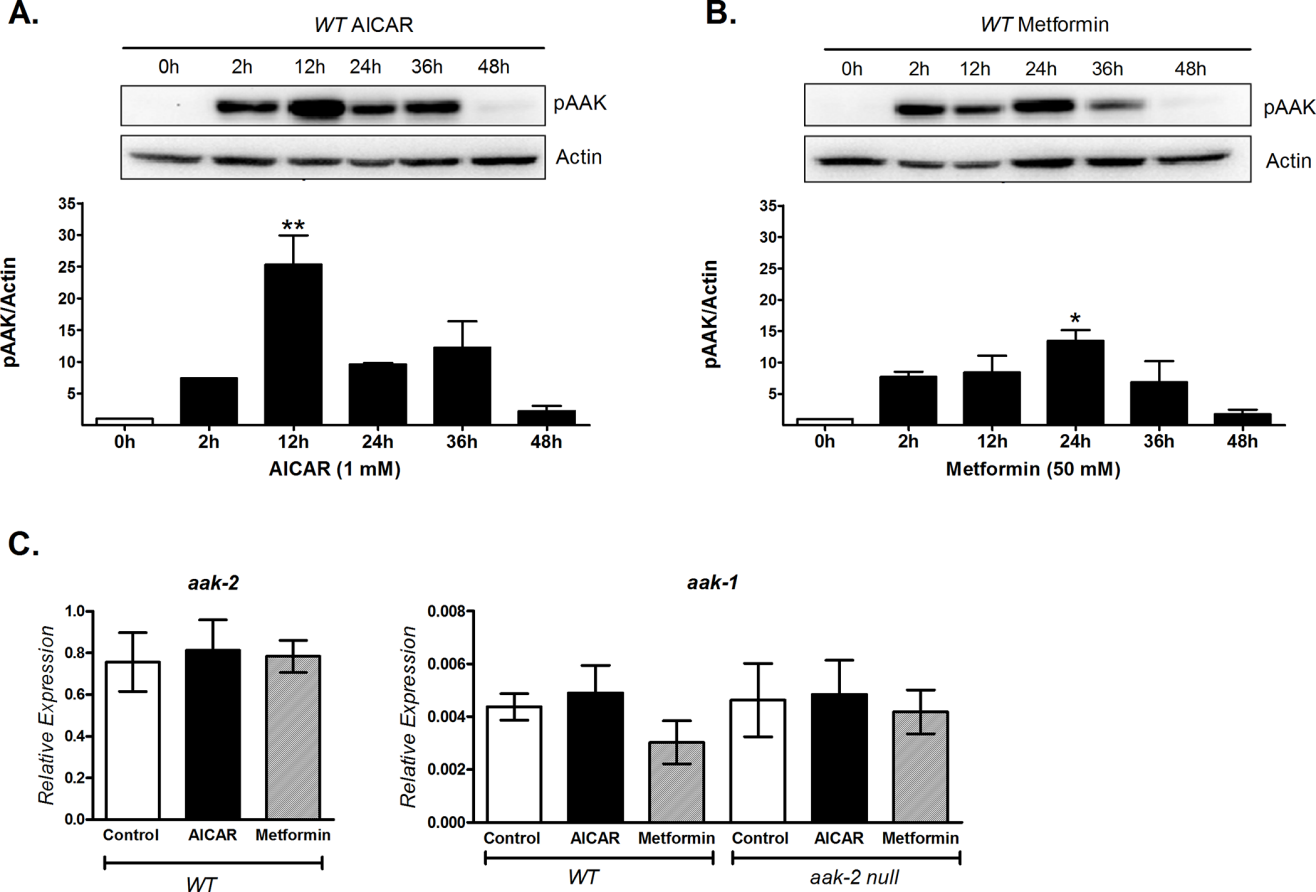
AICAR and metformin increased AAK phosphorylation in a time-dependent manner. AAK phosphorylation at Thr243 (equivalent to Thr172 of mammals) was detected using an anti-pAMPK antibody in N2 adult nematodes treated with A) 1 mM AICAR or B) 50 mM metformin. In both cases, the top panel shows a representative Western blot of phosphorylated AAK (pAAK), where the intensity of the bands represents the phosphorylation level, corresponding to kinase activation. C) Expression levels of *aak-2* and *aak-1* by qRT-PCR in N2 and *aak-2/ok524* strains exposed to 1 mM AICAR for 12 h or 50mM Metformin for 24 h. The graphs show the mean ± SEM of three independent experiments *p<0.05, **p<0.01 vs. 0 h, one-way ANOVA with Bonferroni's *post hoc* test using GraphPad Prism.

To determine the role of AAK in the regulation of energy metabolism in *C*. *elegans*, we first assessed whether it is susceptible to phosphorylation (activation) by two standard activators of AMPK (AICAR or metformin) [25]. Adult wild-type N2 nematodes were exposed to AICAR or metformin at different times (2, 12, 24, 36 and 48 hours). We found that AAK phosphorylation increased in a time-dependent manner with both activators, being earlier and more pronounced with AICAR than with metformin (12 versus 24 h, respectively, Fig 1A and 1B). Hence, 12 and 24 h with AICAR or metformin were used in all subsequent experiments to determine the effect of AAK phosphorylation upon oxidative metabolism. To exclude that the observed surge in AAK phosphorylation during exposure to these activators was due to increased expression of the AAK protein, we measured mRNA expression of the *aak-1* and *aak-2* genes in wild-type N2 animals exposed to AICAR or metformin. Our results showed no differences in the expression levels of these genes (Fig 1C). Even more, in the AAK-2 null mutant (*ok524*) no changes in gene expression of the remaining subunit AAK-1 were observed (Fig 1C). These results demonstrate that both AICAR and metformin increased AAK phosphorylation in *C*. *elegans* in a time-dependent manner without altering protein expression.

### Energy metabolism is changed by AAK phosphorylation in *C*. *elegans*

**Fig 2:**
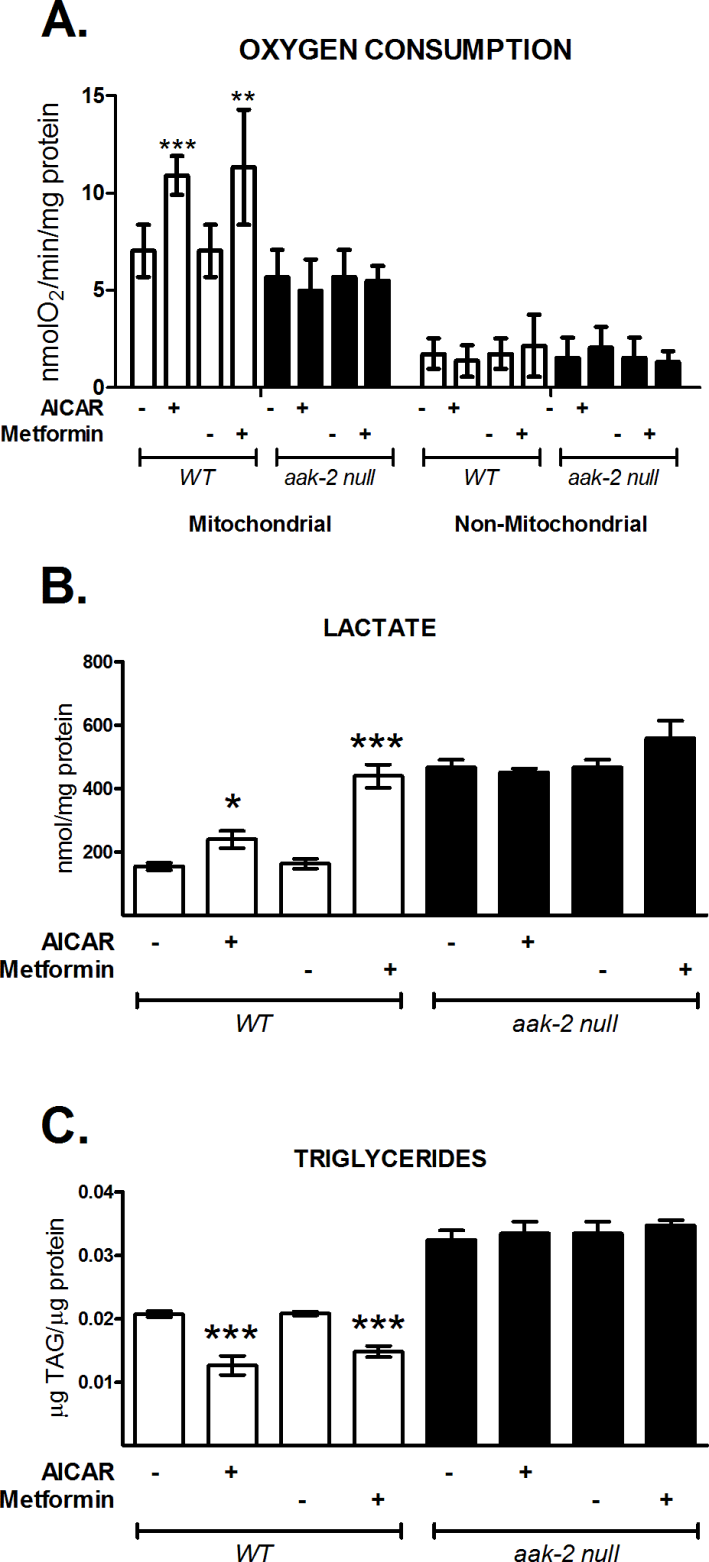
AAK activation by AICAR and metformin enhances oxidative metabolism in *C*. *elegans*. N2 (white box) and *aak-2*/ok524 (black box) nematode strains were grown to the adult stage and exposed to 1 mM AICAR for 12 h or 50 mM metformin for 24 h. A) Mitochondrial and non-mitochondrial oxygen consumption. B) Lactate concentration and C) Total triglyceride content. Each graph shows the mean ± SEM of three independent experiments. The *aak-2* mutant groups showed no change between them. *p<0.05, **p<0.01, ***p<0.001 vs. untreated group, one-way ANOVA with Bonferroni's *post hoc* test, using GraphPad Prism.

Because AMPK activation regulates metabolic flux of nutrients such as carbohydrates and lipids [26], we determined the effect of AAK activation on some energy metabolic markers, such as azide-sensitive oxygen consumption (which is an index of mitochondrial respiration), lactate production and total triglycerides content. We found that the mitochondrial oxygen-consumption rates (Fig 2A) and concentration of lactate (Fig 2B) significantly increased, whereas triacylglycerol (TAG) content decreased (Fig 2C) in N2 wild type nematodes with their AAK activated by AICAR or metformin. To explore whether these changes depended on the AAK-2 subunit, we exposed a null mutant strain of AAK-2 [12] to the AAK activators AICAR or metformin. We found no changes on the physiological outcomes in the *aak-2* mutant strain (Fig 2A–2C). The decreased TAG concentrations could be in line with the increased oxidative metabolism due to AAK activation, as reflected by higher respiration, suggesting that AAK-2 is necessary to promote oxidative metabolism by AICAR and metformin in *C*. *elegans*.

### AAK activation modifies fatty acid composition in *C*. *elegans*

**Fig 3:**
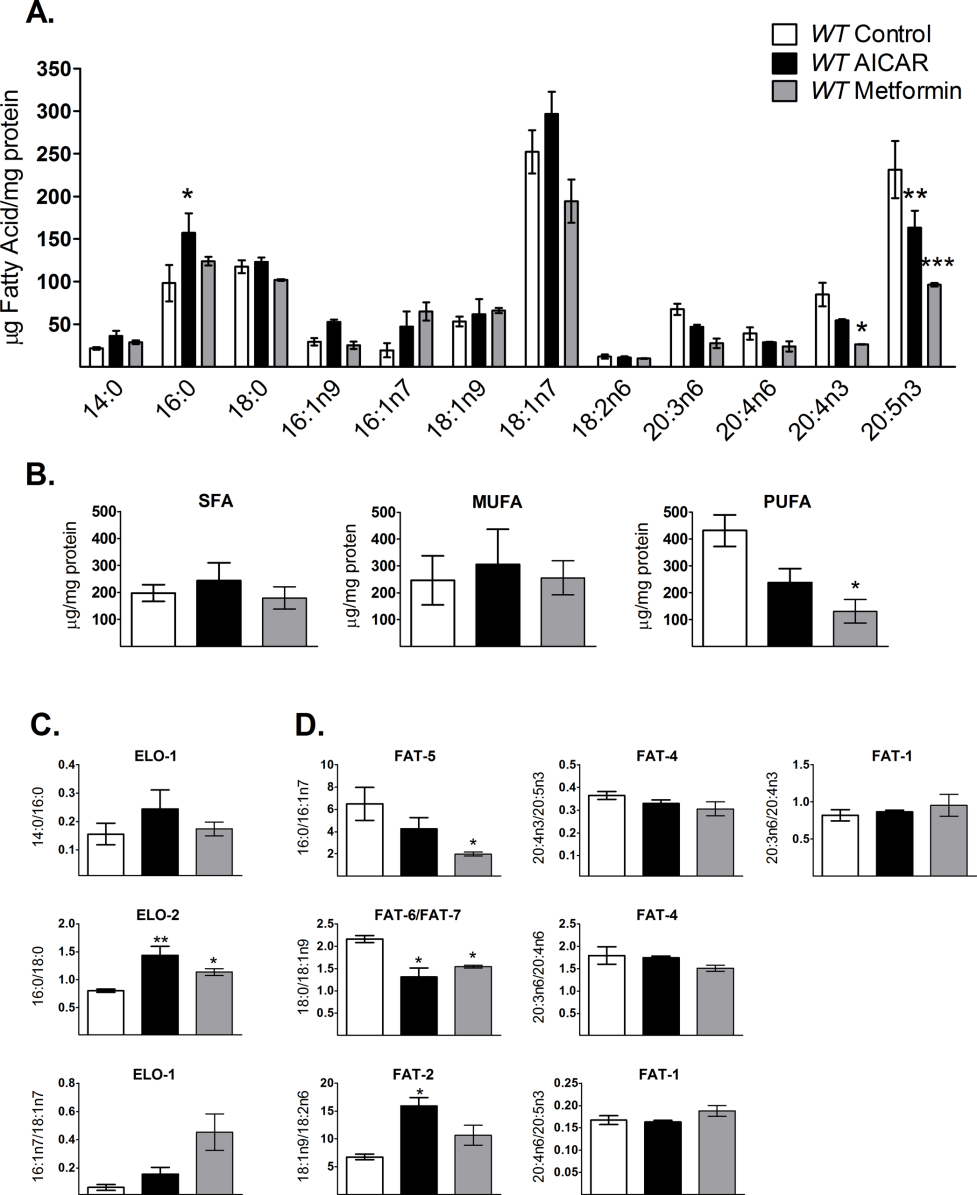
AICAR and metformin modify the fatty acid profile in *C*. *elegans*. Individual fatty acids were determined by gas chromatography analysis. Control wild-type (*WT*) N2 nematodes (white box) were grown in 1 mM AICAR (black box) or in 50 mM metformin (gray box). A) Individual fatty acids: 14:0, myristic acid; 16:0, palmitic acid; 18:0, stearic acid; 16:1n9, 7-palmitoleic acid; 16:1n7, 9-palmitoleic acid; 18:1n9, oleic acid; 18:1n7, vaccenic acid; 18:2n6, linoleic acid; 20:3n6, dihomo-γlinolenic acid (DGLA); 20:4n6, arachidonic acid; 20:4n3, eicosatetraenoic acid; 20:5n3, eicosapentaenoic acid (EPA). B) Fatty acids grouped as saturated (SFA), monounsaturated (MUFA) or polyunsaturated (PUFA) fatty acids. C) Elongase activity indirectly measured from substrate/product ratios. D) Desaturase activity indirectly measured from substrate/product ratios. Each graph shows the mean ± SEM of three independent experiments, *p<0.05, **p<0.01, ***p<0.005 vs. untreated group, one-way ANOVA with Bonferroni's *post hoc* test using GraphPad Prism.

Since reduction of TAG content seems to depend on AAK activation and because TAGs are synthesized depending on fatty acid availability [27], we hypothesized that its activation may change total fatty-acid composition in the worm. Thus, we measured the amount of individual fatty acids in wild-type N2 nematodes after AAK activation and grouped them into saturated fatty acids (SFA), monounsaturated fatty acids (MUFAs) and polyunsaturated fatty acids (PUFAs) (Fig 3A and 3B). Although there were no statistically significant changes in the individual fatty acids; palmitic (C16:0), palmitoleic (C16:1n7), 7-palmitoleic acid (C16:1n9), vaccenic (C18:1n7) and oleic acid (18:1n9), levels of this fatty acids showed a tendency to augment, most likely as a result of the reduction of TAG synthesis induced by AAK activation. In the other hand, di-homo-γ-linolenic (C20:3n6), arachidonic (C20:4n6), omega-3 arachidonic (C20:4n3) and eicosapentaenoic acids (C20:5n3) were reduced upon AAK activation (Fig 3A). Nevertheless, when we analyzed fatty acids by groups, PUFAs showed a statistically significant decrease when AAK was activated. Assuming that this group is the end point of the fatty acid synthesis pathway, this may reveal the effect of AAK in this metabolic route (Fig 3B).

Given that the synthesis of PUFAs involves enzyme-mediated fatty acyl desaturation and elongation [22,28,29], it is possible that some steps in this pathway were modified by activation of AAK. We evaluated individual steps on lipid synthesis by indirectly estimating some desaturase (FAT) and elongase (ELO) activities via their substrate/product ratios from fatty acid profiles [8,30,31]. We found that the ratios of myristic/palmitic (14:0/16:0) and palmitoleic/oleic (16:1n7/18:1n7) have a tendency to increase while palmitic/stearic (16:0/18:0) and oleic/linoleic acids (18:1n9/18:2n6) increased significantly upon AAK activation (Fig 3C and 3D) in N2 worms, indicating that the related activity of elongases ELO-1 and ELO-2 plus desaturase FAT-2 (Δ12-desaturase) may be also affected by phosphorylated AAK. Conversely, FAT-5 and FAT-6/7 desaturases, required for the synthesis of MUFAs, were increased by AAK activation, as shown by palmitate/palmitoleic (16:0/16:1n7) and stearic/oleic (18:0/18:1n9) ratios, while no changes were observed for the ratios representing desaturases of 20-carbon fatty acids, FAT-1 (20:4n6/20:5n3 and 20:3n6/20:4n3) and FAT-4 (20:4n3/20:5n3 and 20:3n6/20:4n6) (Fig 3D). Altogether our data suggest that pharmacological AAK activation decreases the elongation steps and blocks the synthesis of PUFAs; mainly by reducing FAT-2, the rate-limiting enzyme in the conversion of MUFAs to PUFAs in *C*. *elegans* [29].

### NHR-49 and MDT-15 expression is dependent of AAK activation in *C*. *elegans*

**Fig 4:**
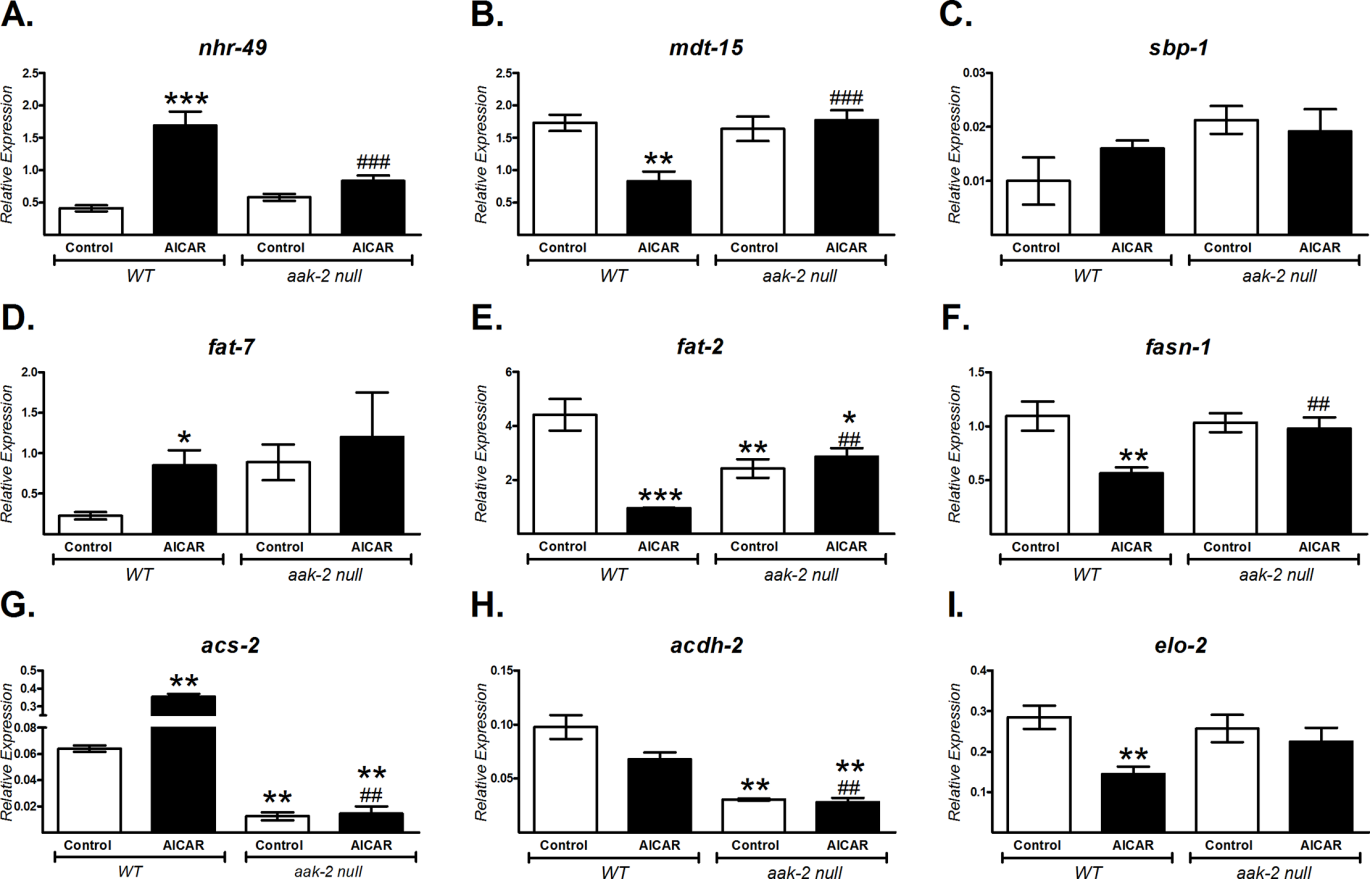
AAK activation-dependent changes of selected transcripts involved in energy metabolism regulation in *C*. *elegans*. Gene expression analysis was performed using qRT-PCR on wild-type N2 (*WT*) and *aak-2*/*ok524* nematodes grown until adults and exposed to 1 mM AICAR for 12 h. A) *nhr-49*, B) *mdt-15*, C) *sbp-1*, D) *fat-7*, E) *fat-2*, *F) fasn-1*, *G) acs-2*, *H) acdh-2 and I) elo-2*. The relative expression of each gene was normalized to endogenous 18S rRNA gene expression. Data shown are the mean ± SEM of three independent experiments, *p<0.05, **p<0.01, ***p<0.001 vs. *WT* control and ^##^p<0.01, ^###^p<0.001 vs. *WT* AICAR, one-way ANOVA with Bonferroni's *post hoc* test using GraphPad Prism.

In mammals, AMPK regulates energy metabolism by modifying the expression of several transcription factors, such as SREBP, PPARα and PGC-1α [10,24]. The *C*. *elegans* orthologues: SBP-1, NHR-49 and MDT-15 respectively, also participate in energy metabolism [6–8]. To study whether AAK acts in a similar manner in the worm, we determined the mRNA expression of *sbp-1*, *nhr-49* and *mdt-15* in wild type animals treated with AICAR, as well as some of their target genes (*fat-7*, *acs-2*, *fat-2*, *acdh-2*, *fasn-1* and *elo-2*) [6–8]. We found that the mRNA expression of *nhr-49* increased and *mdt-15* decreased, whereas *sbp-1* levels did not change (Fig 4A–4C). Surprisingly, although *sbp-1* mRNA levels did not change, the expression of its transcriptional target genes, fatty-acid synthase (*fasn-1*) and elongase (*elo-2)*, decreased significantly in response to AAK activation (Fig 4F and 4I). For *nhr-49*, its own expression and those of its target genes, *fat-7* (Δ9-desaturase) and *acs-2* (acyl-CoA synthetase) increased (Fig 4D and 4G). Conversely, expression of *mdt-15* mRNA as well as its targets *fat-2* (Δ12-desaturase) and *acdh-2* (acyl-CoA dehydrogenase) was diminished (Fig 4E and 4H). These changes were most probably AAK-2-dependent given that we did not observe the increase evoked by AICAR in the mRNA levels of all evaluated genes in the mutant AAK-2 strain (*aak-2*/*ok524*) (Fig 4). Indeed, the lack of AAK-2 significantly decreased expression of *acs-2* and *acdh-2* (Fig 4G and 4H), suggesting the role of this protein in the catabolic pathway of lipids.

### NHR-49 and MDT-15 are involved in the regulation of metabolic changes due to AAK activation

**Fig 5:**
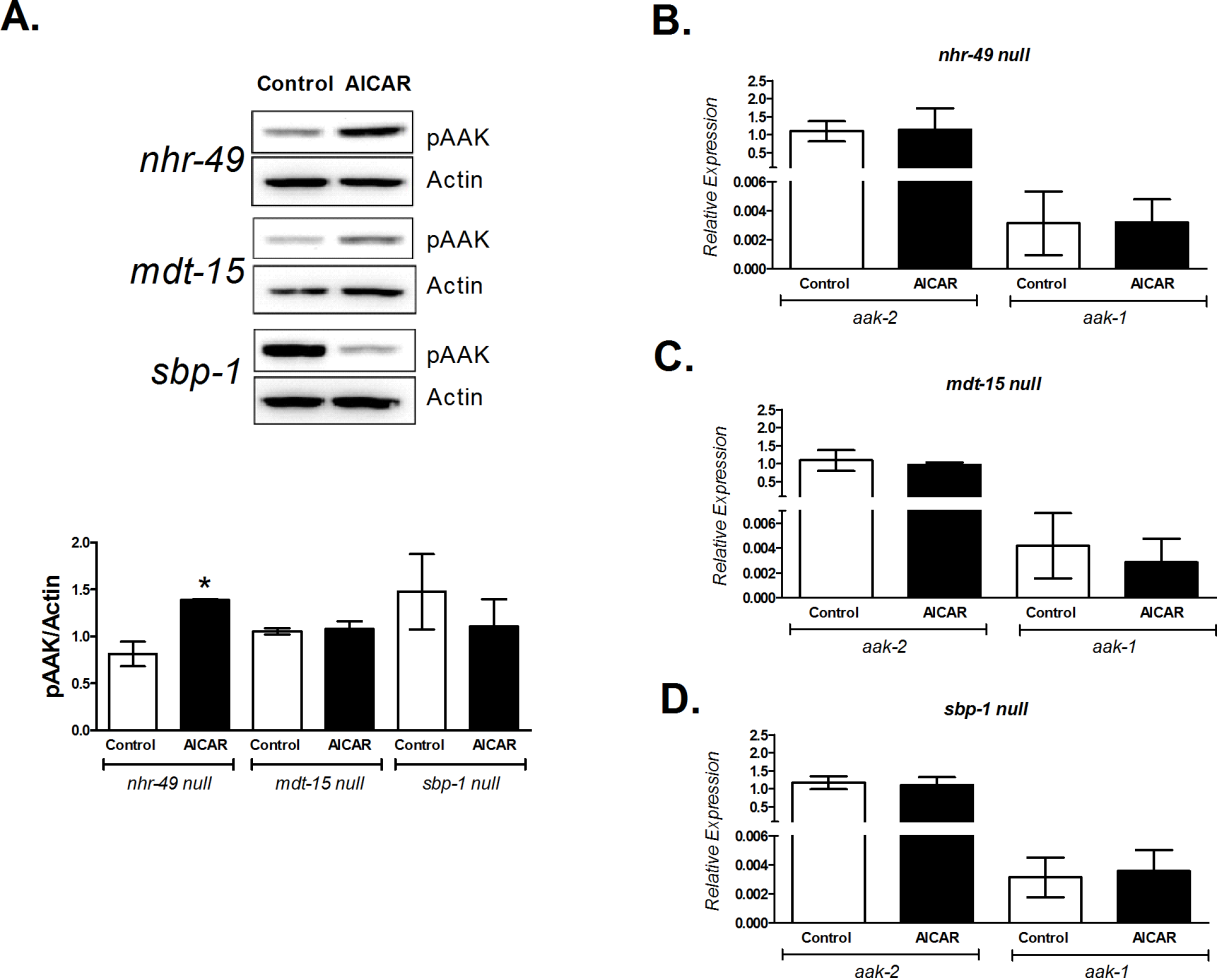
AICAR modifies AAK phosphorylation in mutant strains lacking NHR-49, MDT-15 or SBP-1 transcription factors without altering the expression of both *aak-2* and *aak-1*. Adult worms of the mutant strains *nhr-49/ok2165*, *mdt-15/tm2182* and *sbp-1/ok2363* lacking the proteins NHR-49, MDT-15 and SBP-1, respectively, were exposed to 1 mM AICAR for 12 h. A) AAK phosphorylation at Thr243; top panel shows a representative Western blot of phosphorylated AAK (pAAK), the graph represents the densitometric analysis. B-D) Relative expression of *aak-2* and *aak-1* genes normalized to endogenous 18S rRNA gene expression. Each graph shows the mean ± SEM of three independent experiments, **p<0.01 vs. untreated group of its own strain, one-way ANOVA with Bonferroni's *post hoc* test using GraphPad Prism.

**Fig 6:**
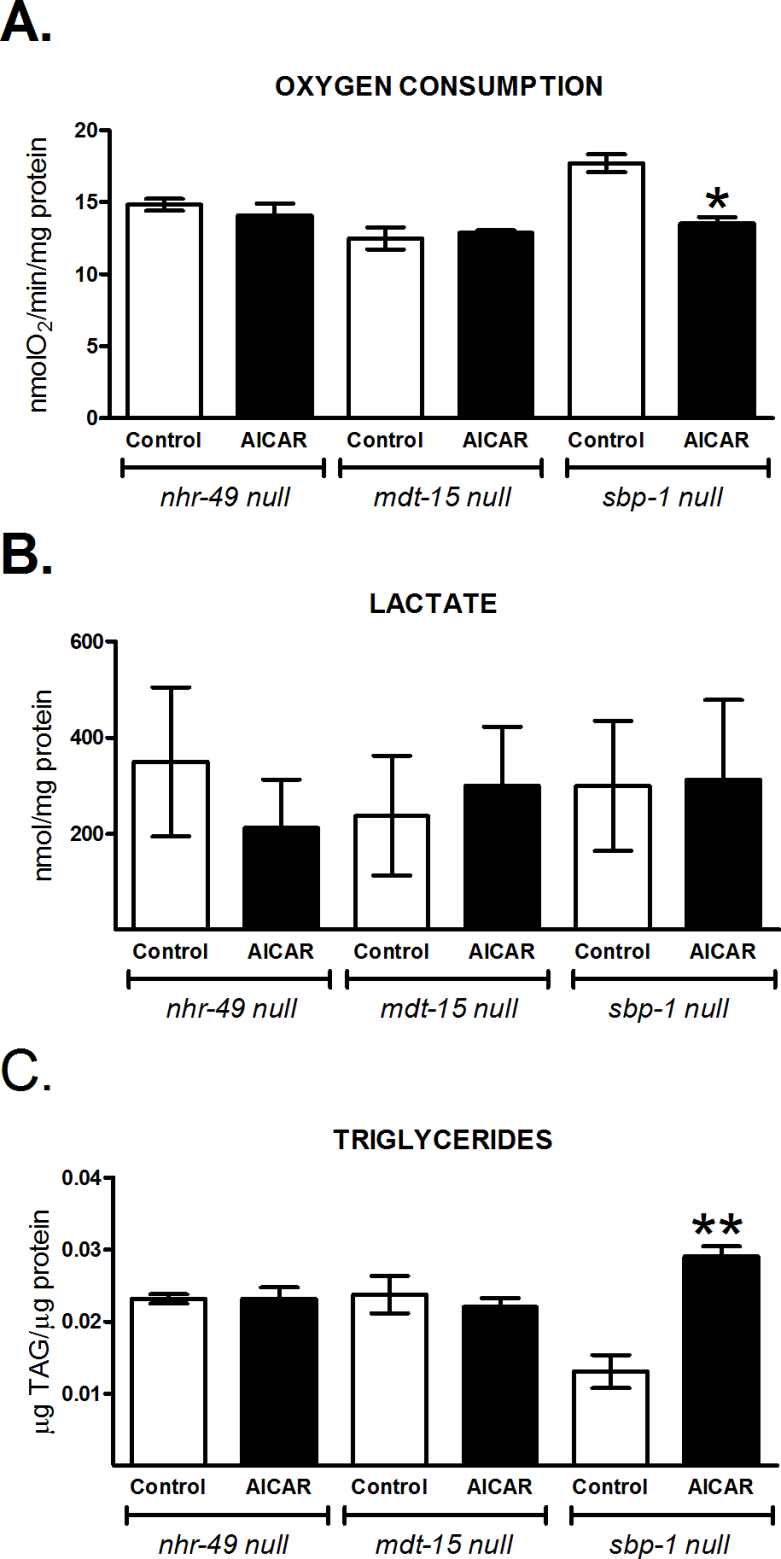
NHR-49, MDT-15 and SBP-1 are required to modulate some metabolic markers upon AICAR exposure. Adult worms of the mutant strains *nhr-49/ok2165*, *mdt-15/tm2182* and *sbp-1/ok2363* lacking the proteins NHR-49, MDT-15 and SBP-1, respectively, were exposed to 1 mM AICAR for 12 h. A) Oxygen consumption rate. B) Lactate concentration and, C) Triglyceride content. Each graph shows the mean ± SEM of three independent experiments, **p<0.01 vs. untreated group of its own strain, one-way ANOVA with Bonferroni's *post hoc* test using GraphPad Prism.

The above results showed that oxidative metabolism is increased by AAK activation in N2 nematodes. To assess if these effects were mediated by the transcriptional factors NHR-49, MDT-15 and SBP-1, we exposed three null mutant strains: *nhr-49* (*ok2165*), *mdt-15* (*tm2182*) and *sbp-1* (*ok2363*) to AICAR and determined AAK phosphorylation, oxygen consumption, lactate concentration and triglyceride content. As shown in Fig 5A, AICAR significantly activates AAK even when NHR-49 is lacking. The absence of *nhr-49*, *mdt-15* or *sbp-1* in the worm did not alter the expression of the catalytic subunits of AAK (*aak-1* and *aak-2*) (Fig 5B, 5C and 5D). Furthermore, in null mutant *nhr-49* and *mdt-15* no changes were observed in oxygen consumption, lactate and triglyceride content (Fig 6A–6C). Conversely, in the *sbp-1* mutant strain we observed a decrease in AAK phosphorylation, although not significant, while lactate did not change and oxygen consumption and TAGs were diminished and increased respectively. Taken together, the above results suggest that activated AAK might modulate oxidative metabolism through these transcription factors.

### Effects of high glucose availability are reversed by AAK activation in *C*. *elegans*

**Fig 7:**
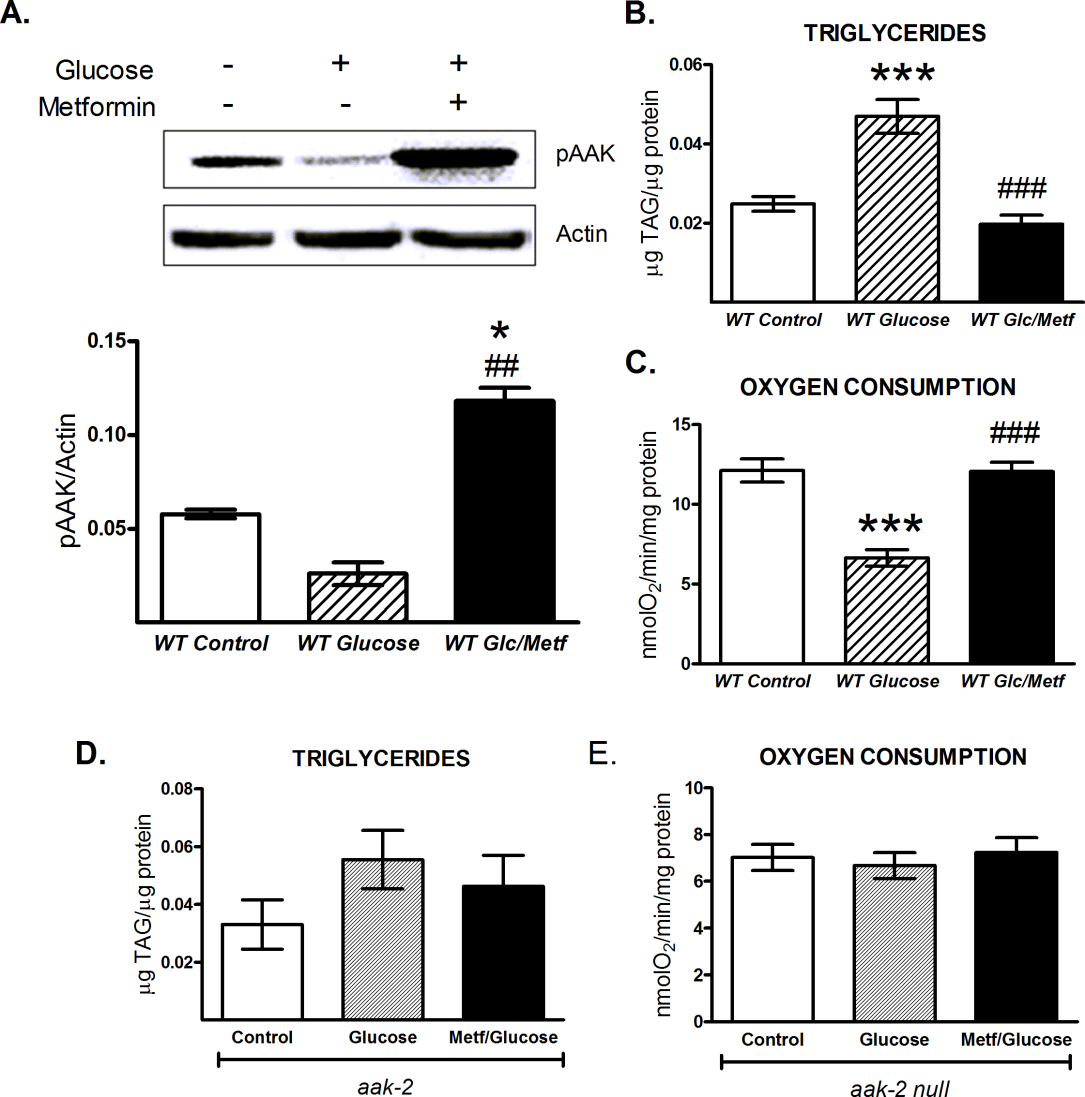
Metformin reverses the deleterious effects of dietary glucose in *C*. *elegans* in an AAK-dependent manner. A-C) Adult wild-type (WT) or D-E) *aak-2/ok524* nematodes were grown on 100 mM glucose with or without 50 mM metformin for 24 h. A) the top panel is a representative Western blot of AAK phosphorylated (pAAK) at Thr243, where the intensity of bands represents the phosphorylation level, corresponding to kinase activation. B and D) triglyceride content and C and E) oxygen consumption rates. The graphs represent the mean ± SEM of three independent experiments, *p<0.05, ***p<0.001 vs. *WT* control and ^##^p<0.01, ^###^p<0.001 vs. *WT* glucose, one-way ANOVA with Bonferroni's *post hoc* test using GraphPad Prism.

Metformin is a first-line drug for treating type 2 diabetes; its proposed mechanism of action involves the AMPK activation [32]. In mammals, chronic exposure to high glucose negatively regulates this kinase [33,34]. To evaluate whether similar effects occur in *C*. *elegans*, we exposed wild-type N2 nematodes to a high glucose concentration (100 mM) in the presence or absence of metformin (50 mM, 24 h). We observed that basal AAK phosphorylation decreased upon glucose administration with respect to control animals, whereas treatment with both glucose and metformin significantly increased AAK phosphorylation even more than control nematodes (Fig 7A). In accordance with a previous report [17], we also found that high glucose provision induced TAG accumulation and decreased the respiratory rate in *C*. *elegans* (Fig 7B and 7C). Since we demonstrated that AAK activation decreased TAG content in N2 wild type worms, it is plausible that AAK activation also modifies TAG accumulation plus oxidative metabolism and prevents deleterious effects when the worms are exposed to a high glucose concentration. As was expected, AAK activation returned TAG content and oxygen consumption to control levels, even in presence of higher glucose availability (Fig 7B and 7C). Altogether, our results suggest that metformin could reverse the effects generated by high glucose availability in an AAK-dependent manner, since such changes were not observed in the *aak-2* mutant strain exposed to high glucose or high glucose plus metformin (Fig 7D and 7E).

## Discussion

Here, we demonstrate that AAK, the *C*. *elegans* AMP-activated protein kinase, can be activated pharmacologically and this modifies energy metabolism by increasing oxidative flux, as indicated by the increase in lactate levels and oxygen consumption. AAK activation by phosphorylation promotes lipid mobilization and decreases PUFAs synthesis. We propose that AAK, NHR-49 and MDT-15 form a regulatory system that coordinates systemic adaptation to energy changes, which in turn, ensures maintenance of homeostasis and health. These data are consistent with the widespread role of AMPK in metabolic control, suggesting that its activation is executed via a similar mechanism from nematodes to humans.

### AAK activation by AICAR and metformin promotes oxidative metabolism in *C*. *elegans*

The fact that the AMPK energy sensor is evolutionarily conserved [10,35], suggests that it may also play an important role in controlling energy metabolism in *C*. *elegans*. To test this idea, we first activated AAK by AICAR or metformin exposure. The concentrations of AICAR and metformin used in our experiments were sufficient to increase AAK activation over the time course evaluated, being faster and more pronounced by AICAR. This fact may reflect a positive allosteric effect of the ZMP, the phosphorylated form of AICAR, similar to AMP, in the worm kinase, which takes place with maximal phosphorylation at 12 h; whereas with metformin this effect was reached until 24 hours of exposure, consistent with the accepted consensus that metformin indirectly activates AMPK [32]. Nevertheless, we cannot exclude the possibility that AICAR has a different potential to activate AAK in comparison with metformin, which would need further experimental exploration.

It is well known that AMPK activation reestablishes cellular energy in part by activation of oxidative metabolism [9]. Consistently, we observed an increase in lactate production and mitochondrial oxygen consumption, but a decrease in lipid storage after AAK activation (Fig 2); suggesting an increased glycolytic and lipolytic flux, as well as augmented β-oxidation, which could explain the observed higher respiration rates and hence, the elevated oxidative metabolic flux. Under AAK activation both, glycolysis and TCA cycle are enhanced at independent ways; lactate production may be increased by effect on rate-limiting glycolytic enzymes hexokinase and phosphofructokinase I and II [36,37], whereas TCA cycle and oxidative phosphorylation may be stimulated by augmenting beta-oxidation that directly supplies acetyl-CoA and reducing equivalents, which in turn reduces pyruvate flux to TCA cycle; in order for glycolysis to proceed, accumulated pyruvate will then rapidly diverge to lactate [36]. Stimulation of the phosphofructokinase I has been shown to increase lactate levels even if aerobic glucose metabolism occurs [38]. The results agree with a recent report where proteomic profiles showed that metformin-treated nematodes have up-regulated expression of several proteins from glycolysis and the tricarboxylic acid cycle (TCA) and corresponding increased respiration rates [39]. Augmented oxygen consumption by metformin treatment *a priori* seems inconsistent with the general idea that metformin inhibits complex I of the mitochondrial electron transport chain and hence decreases respiration [40]. But our data as well as previous reports [39,40], indicate that increased carbohydrate and fatty acid oxidation produce mediators that accelerate the TCA cycle to activate mitochondrial respiration. Indeed, metformin inhibition of complex I is an acute process, whereas the observed result in the worm seems a long term effect. It is noteworthy that fatty acid oxidation cannot occur when mitochondrial respiration is interrupted; supporting the fact that metformin treatment may result in up-regulation of several catabolic processes as a consequence of sustained AAK activation in *C*. *elegans*.

In *C*. *elegans*, the orthologs of the AMPK catalytic subunits α1 and α2 are encoded by the *aak-1* and *aak-2* genes [12]. It has been proposed that AAK-2 participates in longevity, stress resistance and some effects of dietary restriction, whereas mutation of AAK-1 does not cause obvious abnormalities [12,13,15,21,41,42]; suggesting that AAK-2 is the subunit responsible for its kinase activity. Nevertheless, the effects of AAK-2 activation on energetic metabolism in *C*. *elegans* are still unknown. In this sense, our data suggest that AAK-2 primarily modulates energy metabolism, in view of the fact that its activation modifies several metabolic markers such as lactate production, TAGs and oxygen consumption. Accordingly, it was plausible to expect that *aak-2* deletion declines the mitochondrial activity reflected by a reduced-oxygen consumption, which in turn relieves inhibition of glycolytic flux and promotes lactate accumulation, even more than in AAK activated state. Consistently, in this paper we found that in animals carrying *aak-2* deletion, lactate production is more increased than in wild type animals with the AAK activated (Fig 2B), while mitochondrial oxygen consumption rate is decreased (Fig 2A).

Moreover, in nematodes lacking the AAK-2 protein (*aak-2* null, ok524) metformin or AICAR failed to change the mentioned catabolic fluxes (Fig 2), supporting that the fuel-conserving role of AMPK is evolutionarily conserved.

### Metabolic changes triggered by AAK are dependent on NHR-49, MDT-15

Because AAK activation diminishes fatty-acid storage, it is possible that this regulation might be mediated by transcription factors as occurs in mammals. We observed that the NHR-49, MDT-15 and SBP-1 transcription factors are involved in the effects produced by AAK activation, such as reduced total TAGs and PUFAs (Figs 2C and 3A), suggesting a possible link between AAK activity and these metabolic molecules.

The *C*. *elegans* functional orthologue of mammalian PPARα, NHR-49, is a key regulator of fat mobilization. This factor couples β-oxidation and desaturation of lipids by regulating several enzymes such as ACS-2 (acyl-CoA synthetase), CPT-5 (carnitine palmitoyltransferase), ECH-1 (enoyl-CoA hydratase) and those with Δ9-desaturase activities (FAT-5, FAT-6 and FAT-7) [22,23] that can stimulate mitochondrial respiration. We found that AAK activation increased gene expression of *nhr-49*, *fat-7 and acs-2* (Fig 4A, 4D and 4G), as well as augmenting Δ9-desaturase activities (Fig 3D); suggesting that the observed reduction of TAGs and increase of oxygen consumption were probably mediated by NHR-49, since no changes were observed in the mutant *nhr-49* strain treated with AICAR (Fig 6A and 6C). Moreover, in the mutant lacking AAK-2, expression of *acs-2* was severely depressed (Fig 4G), indicating the participation of this component in the reducing effect of AAK activation on TAG content (Fig 6C), as well as the link between AAK and NHR-49 to modulate lipid oxidation through ACS-2. These findings are in agreement with the function of mammalian PPARα, which driven by AMPK is able to modulate enzymes involved in lipid catabolism [43,44].

Like NHR-49, MDT-15 is also vital for metabolic control by activating gene expression of *acdh-1*/*2* and *fat-2*, which encode a short-chain acyl-CoA dehydrogenase and a Δ12-fatty acyl desaturase respectively, related to β-oxidation and lipid synthesis [8]. FAT-2 desaturase controls the conversion of MUFAs to PUFAs by catalyzing the first limiting step of PUFAs synthesis [29]. Our data showed diminished *mdt-15* and *fat-2* gene expression (Fig 4B and 4E), as well as reduced FAT-2 desaturase activity (Fig 3D) and lower amounts of 18:2n6 and C20 PUFAs (Fig 3A and 3B) after AAK activation. By contrast, SFA such as palmitic and stearic acids remained with slight or no changes (Fig 3A). This can be explained at least in part, because more than 90% of palmitic acid is provided to *C*. *elegans* from the bacteria in the diet [4,45]. On the other side, enhanced beta-oxidation by activation of AAK may contribute to keep palmitic acid and other SFA at steady state levels.

However, no changes were observed in the *mdt-15* null mutant upon AAK activation (Figs 5 and 6); suggesting that MDT-15, as NHR-49, is also involved in fatty acid desaturation and β-oxidation to maintain lipid homeostasis. MDT-15 in *C*. *elegans* has been reported to serve the same function as the mammalian PGC-1α [8]. This gene is up regulated by AMPK [46,47], but we did not observe an increase in MDT-15 expression under our conditions of AAK activation (Fig 4B). Nevertheless, the intriguing decrease of MDT-15 that we observed could ensure the appropriate gene expression to cooperate with the metabolic effects orchestrated by AAK activation.

Moreover, as in mammals, lipid synthesis in *C*. *elegans* is also mediated by the SREBP homolog SBP-1 by augmenting the transcription of genes coding for lipogenic enzymes, such as *elo-2*, *acc-1* and *fasn-*1 that drive fat accumulation and adipogenesis [6,48]. Although *sbp-1* expression did not change by AAK activation; its target genes, *fasn-1* and *elo-2*, as well as the estimated activity of elongase ELO-2 significantly decreased (Fig 4F and 4I). ELO-2 is responsible for the elongation of palmitic acid and PUFA biosynthesis [28]. Consistently, we observed a slight increase in palmitate and a clear diminution of PUFAs (Fig 3A and 3B). As mentioned before, palmitic acid in *C*. *elegans* comes primary from the diet [4,45]. By contrast, PUFA are not available in the main source of food for *C*. *elegans* when cultured in the laboratory (*E*. *Coli* OP50) then, PUFA found in the nematode come from *de novo* synthesis [4,45]. Thus, the high availability of palmitic acid probably maintains its level near to the steady state, in part because of its participation in the biosynthesis of MUFA mediated by FAT-6/7, although FAT-2 is hindered. As well, AMPK-stimulated β-oxidation may contribute to the balance of palmitic acid and other SFA levels. Since the Δ12 desaturase is the first step in the synthesis of PUFA, strains lacking this activity would be expected to be devoid of PUFA. Indeed, our data showed diminished *fat-2* gene expression (Fig 4E), as well as reduced FAT-2 desaturase activity (Fig 3D) after AAK activation. It is conceivable that if the rate-limiting step for the synthesis of PUFA is decreased, no changes will be reflected in MUFAs, even if the activity of FAT-7 increases.

Although mammalian liver SREBP is inhibited by phosphorylation upon AMPK activation [49], this posttranslational modification has not yet been demonstrated in *C*. *elegans*; thus, our results suggest that a similar event may occur in the worm by AAK activation. Besides, it was shown that SREBP1c regulates expression of the enzyme that elongates C18-20 for PUFA biosynthesis (Elovl5) [50]. Interestingly, miRNAs, emerging as important metabolic regulators, in *C*. *elegans* have been implicated in the control of lipid metabolism. In fact, it has been shown that miR-786 directly represses *elo-2* expression resulting in significantly elevated palmitate levels [51]. Although to our knowledge no data for *fasn-1* is available, we cannot exclude the possibility that it is also regulated in a similar fashion, because miR-122 affects the expression of the mammalian *fasn-1* orthologue, fatty acid synthase (*FAS)* [52]. Taken together, the data enables us to suggest that similar miRNA mechanisms might operate in our model. Thus, the apparent routing between MDT-15, NHR-49 and likely SBP-1, might explain the increased oxidative metabolism, as a result of the enhanced glycolytic and lipolytic rates that feed β-oxidation, which could operate as substrate sources for the mitochondrial respiratory chain. Additionally, determining changes of fatty acid profiles in AAK activator-treated nhr-49/ok2165, mdt-15/tm2182 and sbp-1/ok2363 mutant strains, would be interesting for deep knowledge about the AAK axis in the metabolic control in *C*. *elegans*.

### AAK activation by metformin reverses the effects evoked by high glucose diet in *C*. *elegans*

Glucose is an energy molecule and its systemic levels are actively regulated, such that disruption of its metabolism can lead to human diseases like obesity and type 2 diabetes. In this sense, a high-glucose media has deleterious effects on *C*. *elegans*, suggesting altered metabolic homeostasis [53,54]. In our study, we observed that nematodes exposed to high glucose exhibited increased TAG content, diminished mitochondrial oxygen consumption and decreased AAK phosphorylation. Given the important role of AMPK as a “fuel gauge” [9,10] plus a strong link between low AMPK activation and a state of over-nutrition [55], it is plausible that a dysfunction in the AAK signaling pathway by a carbohydrate-rich diet may result in systemic perturbations that contribute to impaired mitochondrial metabolism, as previously suggested [17]. Following the enhanced effect of metformin on the oxidative fluxes in *C*. *elegans*, we wondered whether this drug was able to reverse the metabolic disruption evoked by high glucose exposure in *C*. *elegans*. Strikingly, we showed that in wild-type nematodes metformin prevented the effects generated by excess glucose intake, restoring the metabolic markers to control levels; similar to what occurs in obese and diabetic patients, where chronic metformin treatment decreases glucose and lipid levels in plasma [56]. In contrast, growing *aak-2* mutant nematodes on high glucose and metformin did not produce any metabolic changes (Fig 7D and 7E), implying that AAK is a cellular metabolic checkpoint responding to an altered energy status, such as high glucose feeding.

In sum, we show the role of the energy sensor AAK in the metabolic control of *C*. *elegans*, likely through the AAK/NHR-49/MDT-15 axis. This axis is an integral part of a regulatory mechanism that coordinates systemic adaptation to energy challenges, which ensures and maintains energy homeostasis and hence promotes a healthy state. In addition, this work provides evidence that supports a relationship between nutritional perturbations and transcriptional factors that trigger mechanisms to restore energy homeostasis in an AAK-dependent fashion in *C elegans*. Fatty acid and carbohydrate metabolism are complex processes that are highly associated with pathologies such as diabetes or obesity. Additionally, many gaps in knowledge still exist, partly due to the lack of *in vivo* research concerning energy metabolism regulation. Thus, *C*. *elegans* is potentially an exciting screening model of metabolic signaling pathways to discover new therapeutic targets in pathologies associated with energy alterations.

## Materials and Methods

### Nematode strains and growth conditions

*C*. *elegans* strains N2-Bristol (wild-type), *aak-2/ok524*, *sbp-1/ok2363*, *mdt-15/tm2182* and *nhr-49/ok2165* were obtained from the Caenorhabditis Genetics Center (CGC, University of Minnesota, Minneapolis, MN) strain bank. *C*. *elegans* growth was done according to standard laboratory protocols [57]. Synchronized nematodes were maintained until adulthood in plates with nematode growth medium (NGM) plus 25 μM 5-fluorodeoxyuridine (FUDR) (Sigma) and with *E*. *coli* OP50-1 as the food source. When required, NGM plates were prepared with 100 mM glucose, 1 mM AICAR (Sigma) or 50 mM metformin (Aldrich) and the nematodes were exposed for different times (2, 12, 24, 36 or 48 h), after which excess bacteria were removed with M9 buffer (0.02 M KH_2_PO_4_, 0.04 M Na_2_HPO_4_, 0.085 M NaCl, 1 ml 1 M MgSO_4_, H_2_O to 1 liter) [58], the nematodes were collected, quickly frozen in liquid nitrogen and stored at -70°C until analysis.

### Determination of AAK activation

AAK phosphorylation (activation) was evaluated by Western blot. The nematodes were homogenized mechanically using a mortar and pestle with protein lysis buffer [50 mM HEPES, 50 mM KCl, 1 mM EDTA, 1 mM EGTA, 5 mM β-glycerol phosphate, 0.1% Triton X-100, protease inhibitor cocktail (Complete, Roche), 50 mM NaF, 1 mM sodium orthovanadate, 5 mM sodium pyrophosphate and 0.2 mM PMSF], collected and centrifuged (10,000 g, 10 min, 4°C). The protein concentration was quantified using the Bradford method [59]. 30 μg of protein was subjected to SDS-PAGE and transferred to PVDF membranes. pAAK was identified using an anti-phospho-AMPKα antibody (Cell Signaling Technology). The signal was quantified using chemiluminescent detection and Quantity One 1-D Analysis v4.6.5 image processing software (Bio-Rad). An anti-actin antibody was used as the loading control.

### Lactate and triglyceride quantification

Lactate concentration was determined using the biochemical end point method described previously (based on the Lactate Dehydrogenase assay) [60,61]. Briefly, the nematodes were mechanically homogenized in fresh HClO_4_ 3%/EDTA 20 mM, the supernatant resulting from centrifugation was neutralized in KOH 4N and used immediately to measure lactate. Although values for lactate are reported at nmol/mg protein, we found that 1 mM of lactate is approximately equivalent to 40.4 nmol of lactate/mg protein in one day of adulthood N2 nematodes. Which corresponds to values already reported by others [62].

Triglycerides were essentially quantified as described previously [63]. Briefly, the nematodes were homogenized mechanically in protein lysis buffer and then heated to 90°C for 5 min, followed by vortexing and centrifugation (10,000 g, 10 min, 20°C). The concentration was determined using a TAG assay kit (Sigma-Aldrich) by lipase enzymatic hydrolysis of the triglycerides to glycerol and free fatty acids. Metabolite concentrations were normalized to the total protein concentration of each sample as described before.

### Oxygen consumption rate

The oxygen consumption rate was determined essentially as described previously [61], using a Clark-type oxygen electrode and an YSI 5300A Biological Oxygen Monitor. The living nematodes were collected, washed to remove excess bacteria and re-suspended in 50 μl of M9 buffer. The slopes were recorded for at least 10 minutes at 24°C [64] on a Bio-Rad 1325 Econo-Recorder. The O_2_ solubility value used was 202.94 nmolO_2_/ml at 24°C, 585mmHg and 77% saturation, corresponding to Mexico City altitude [65,66]. Oxygen consumption measurements were done in 1 ml of M9 buffer and normalized to the total protein, which was determined as above described in worms recovered from the oxygen chamber at the end of the measurement. To evaluate the mitochondrial contribution to the oxygen consumption rates of the entire animals, we added 1 mM sodium azide for 5 min after starting the measurement.

### RNA extraction and qRT-PCR

RNA extraction from nematodes was made based on the extraction method with Trizol reagent (Ambion) [67]. cDNA was obtained using random hexamer oligonucleotides and the enzyme M-MLV Reverse Transcriptase (Invitrogen). Expression analyses were performed using Taqman probes for: *nhr-49* (Ce02412666_g1), *mdt-15* (Ce02406577_g1), *sbp-1* (Ce02453004_m1), *fasn-1* (Ce02411653_g1), *fat-7* (Ce02477066_g1), *elo-2* (Ce02469363_g1), *acs-2* (Ce02486191_g1), *acdh-2* (Ce02432820_g1), *aak-2* (Ce02404259_g1) and *aak-1* (Ce02406988_g1) genes. The probes were detected using the StepOne System and StepOn Software v2.2 (ABI instruments). The mRNA levels were normalized to eukaryotic 18S rRNA gene expression as an endogenous control.

### Analysis of total fatty acid profiles

Total fatty acids were extracted from nematodes essentially following a previous method [68] with heptadecanoic acid (internal standard) and chloroform-methanol (2:1, v/v) [69] having 0.002% n-butyl-hydroxy-toluene as an antioxidant to avoid further oxidation [70]. The generation of fatty-acid methyl esters (FAMEs) was done following an earlier method [29] by dissolving the obtained lipid fraction with a mixture of 0.2 mL toluene, 1.76 mL methanol and 0.04 mL H_2_SO_4_ before incubating the samples at 95°C for 1 h. The concentration and composition of FAMEs were evaluated as described previously [71] using a Shimadzu GC-8a gas chromatodph. Helium gas was used as the carrier of the sample and the detection was performed using a H_2_ flame. The chromatograph injector was set at 250°C and the initial temperature of the column ranged from 150 to 220°C (final). The peaks of each fatty acid were quantified by comparison with the concentration of the internal standard.
